# Temporal Patterns of Influenza A and B in Tropical and Temperate Countries: What Are the Lessons for Influenza Vaccination?

**DOI:** 10.1371/journal.pone.0152310

**Published:** 2016-03-31

**Authors:** Saverio Caini, Winston Andrade, Selim Badur, Angel Balmaseda, Amal Barakat, Antonino Bella, Abderrahman Bimohuen, Lynnette Brammer, Joseph Bresee, Alfredo Bruno, Leticia Castillo, Meral A. Ciblak, Alexey W. Clara, Cheryl Cohen, Jeffery Cutter, Coulibaly Daouda, Celina de Lozano, Domenica De Mora, Kunzang Dorji, Gideon O. Emukule, Rodrigo A. Fasce, Luzhao Feng, Walquiria Aparecida Ferreira de Almeida, Raquel Guiomar, Jean-Michel Heraud, Olha Holubka, Q. Sue Huang, Herve A. Kadjo, Lyazzat Kiyanbekova, Herman Kosasih, Gabriela Kusznierz, Jenny Lara, Ming Li, Liza Lopez, Phuong Vu Mai Hoang, Cláudio Maierovitch Pessanha Henriques, Maria Luisa Matute, Alla Mironenko, Brechla Moreno, Joshua A. Mott, Richard Njouom, Nurhayati, Akerke Ospanova, Rhonda Owen, Richard Pebody, Kate Pennington, Simona Puzelli, Mai thi Quynh Le, Norosoa Harline Razanajatovo, Ana Rodrigues, Juan Manuel Rudi, Raymond Tzer Pin Lin, Marietjie Venter, Marie-Astrid Vernet, Sonam Wangchuk, Juan Yang, Hongjie Yu, Maria Zambon, François Schellevis, John Paget, Global Influenza B Study

**Affiliations:** 1 Netherlands Institute for Health Services Research (NIVEL), Utrecht, The Netherlands; 2 Sección de Virus Respiratorios y Exantemáticos, Instituto de Salud Pública de Chile, Santiago de Chile, Chile; 3 Istanbul University, Istanbul, Turkey; 4 National Influenza Center, Ministry of Health, Managua, Nicaragua; 5 National Influenza Center, Institut National d'Hygiène, Ministry of Health, Rabat, Morocco; 6 National Center for Epidemiology, Surveillance and Health Promotion National Institute of Health, Rome, Italy; 7 Epidemiology and Prevention Branch, Influenza Division, Centers for Disease Control and Prevention, Atlanta, GA, United States of America; 8 Centro de Referencia Nacional de Influenza y Otros Virus Respiratorios, Instituto Nacional de Investigación en Salud Pública (INSPI), Guayaquil, Ecuador; 9 National Influenza Center, Ministry of Health, Guatemala City, Guatemala; 10 US Centers for Disease Control, Central American Region, Guatemala City, Guatemala; 11 Centre for Respiratory Diseases and Meningitis (CRDM), National Institute for Communicable Diseases, Johannesburg, South Africa; 12 School of Public Health, Faculty of Health Science, University of the Witwatersrand, Johannesburg, South Africa; 13 Communicable Diseases Division, Ministry of Health, Singapore, Singapore; 14 Respiratory Viruses Unit, Pasteur Institute of Côte d’Ivoire, Abidjan, Côte d’Ivoire; 15 National Influenza Center, Ministry of Health, San Salvador, El Salvador; 16 Public Health Laboratory, Department of Public Health, Ministry of Health, Thimphu, Bhutan; 17 US Centers for Disease Control and Prevention, Nairobi, Kenya; 18 Division of Infectious Disease, Key Laboratory of Surveillance and Early-warning on Infectious Disease, Chinese Center for Disease Control and Prevention, Beijing, China; 19 Ministry of Health, Brasília, DF, Brazil; 20 National Influenza Reference Laboratory, Infectious Diseases Department, National Institute of Health, Lisbon, Portugal; 21 National Influenza Center, Virology Unit, Institut Pasteur of Madagascar, Antananarivo, Madagascar; 22 L.V.Gromashevsky Institute of Epidemiology and Infectious Diseases National Academy of Medical Science of Ukraine, Kiev, Ukraine; 23 Institute of Environmental Science and Research, Wellington, New Zealand; 24 Zonal Virology Laboratory, Astana Center of Sanitary Epidemiology Expertise, Astana, Kazakhstan; 25 US Naval Medical Research Unit No. 2, Jakarta, Indonesia; 26 Instituto Nacional de Enfermedades Respiratorias “Dr. Emilio Coni,” Santa Fe, Argentina; 27 National Influenza Center, Ministry of Health, San José, Costa Rica; 28 National Institute of Hygiene and Epidemiology, Hanoi, Vietnam; 29 National Influenza Center, Ministry of Health, Tegucigalpa, Honduras; 30 National Influenza Center, IC Gorgas, Panama City, Panama; 31 Service de Virologie, Centre Pasteur du Cameroun, Yaounde, Cameroon; 32 Vaccine Preventable Diseases Surveillance Section, Health Policy Protection Branch, Office of Health Protection, Department of Health, Woden, Australia; 33 Respiratory Diseases Department, Public Health England, Colindale, United Kingdom; 34 National Influenza Center, National Institute of Health, Rome, Italy; 35 National Institute of Health, Epidemiology Department, Lisbon, Portugal; 36 Global Disease Detection, US-CDC, Pretoria, South Africa; 37 Zoonoses Research Center, Department of Medical Virology, University of Pretoria, Pretoria, South Africa; 38 Respiratory Virus Unit, Public Health England, Colindale, United Kingdom; Public Health Agency of Canada, CANADA

## Abstract

**Introduction:**

Determining the optimal time to vaccinate is important for influenza vaccination programmes. Here, we assessed the temporal characteristics of influenza epidemics in the Northern and Southern hemispheres and in the tropics, and discuss their implications for vaccination programmes.

**Methods:**

This was a retrospective analysis of surveillance data between 2000 and 2014 from the Global Influenza B Study database. The seasonal peak of influenza was defined as the week with the most reported cases (overall, A, and B) in the season. The duration of seasonal activity was assessed using the maximum proportion of influenza cases during three consecutive months and the minimum number of months with ≥80% of cases in the season. We also assessed whether co-circulation of A and B virus types affected the duration of influenza epidemics.

**Results:**

212 influenza seasons and 571,907 cases were included from 30 countries. In tropical countries, the seasonal influenza activity lasted longer and the peaks of influenza A and B coincided less frequently than in temperate countries. Temporal characteristics of influenza epidemics were heterogeneous in the tropics, with distinct seasonal epidemics observed only in some countries. Seasons with co-circulation of influenza A and B were longer than influenza A seasons, especially in the tropics.

**Discussion:**

Our findings show that influenza seasonality is less well defined in the tropics than in temperate regions. This has important implications for vaccination programmes in these countries. High-quality influenza surveillance systems are needed in the tropics to enable decisions about when to vaccinate.

## Introduction

Despite a lack of influenza surveillance data for many countries, the burden of disease is estimated to be high everywhere that it has been systematically studied, including the tropics [1–5]. Annual vaccination, the cornerstone of influenza prevention and control, however, has not been implemented in all countries, especially those in the inter-tropical belt [6].

Since 1999, the World Health Organization has issued semi-annual recommendations for the composition of influenza vaccines to be used in the Southern and Northern hemispheres [7–8], but they do not currently issue specific recommendations for the inter-tropical belt [9]. The 2012 recommendations of the World Health Organization Strategic Advisory Group of Experts identified the highest priority groups for influenza vaccination in countries with no vaccination programmes [10]. In addition to correct matching of vaccine strains with circulating strains, optimal timing of vaccination is necessary to obtain maximal vaccine effectiveness. This is particularly important as vaccine-induced immunity may wane rapidly after influenza vaccination, especially in the elderly [11]. Whilst seasonal influenza epidemics usually take place in the same period of the year in countries far from the equator, this is not always the case for countries in the tropics, and the timing can vary even between neighbouring countries or countries at the same latitude [9, 12–13].

Approximately one-third of the world’s population lives in the intra-tropical region, and it is thought to have a high burden of influenza disease [1]. Accurate epidemiological data is needed for the prevention and control of influenza in tropical countries, but data are scarce, although this has been changing slowly since the 2009 influenza H1N1 pandemic [14]. In this study, we examined the temporal characteristics of influenza epidemics (overall and separately for influenza A and B) in the Northern hemisphere, Southern hemisphere, and intra-tropical region using data collected by the Global Influenza B Study, and we discuss their potential implications for influenza vaccination programs.

## Materials and Methods

### Study design

We analyzed anonymous influenza surveillance data that were provided to countries participating to the Global Influenza B Study. Consent was obtained, when necessary, at the stage of data collection, i.e. separately from each country. No further permission is required to analyze the data.

This was a retrospective study of epidemiological data in the Global Influenza B Study database, which includes surveillance data from 30 countries [15]. Data include the weekly influenza-like illness/acute respiratory infection consultation rates (per 100,000 population or 100 consultations, depending on the country), the weekly number of respiratory specimens tested for influenza viruses, and the weekly number of influenza-positive specimens (“cases”) by age and by virus type, subtype, and lineage. Except in the USA (until 2002), Italy, Kazakhstan, Morocco, Portugal, Turkey, and Ukraine, where virological data are not available for summer months (approximately week 20–40), data are available for all weeks of the year in all countries. Data stratified by region are available for China (north and south) and for Brazil (north, northeast, central-west, southeast, and south) [16]. Information was also collected on the main features of the national influenza surveillance system in each participating country [15].

### Statistical analysis

The unit of analysis was the “season”, which corresponded to the calendar year for countries in the Southern hemisphere and the inter-tropical belt and to week 27 of a given year to week 26 of the following year for countries in the Northern hemisphere. Separate analyses were performed for the two regions of China and the five regions of Brazil. For the analyses, “country” was defined as the country or, for China and Brazil, as the country region. The location of each country was determined (Southern hemisphere, Northern hemisphere, or inter-tropical belt) according to the location of country centroid or, if not available, the country’s largest city [17]. The pandemic season 2009 (2009–2010 in the Northern hemisphere) was excluded from the analysis because the timing of the influenza epidemics was unusual and could not be compared to the other seasons. In addition, all seasons with fewer than 50 reported influenza cases were excluded from the analysis [18].

The percentage of laboratory-confirmed influenza cases was calculated for each month of each season. For this analysis, a week was considered to be in a given month if four or more days of it were in the month. The primary peak was defined as the week with the most reported influenza cases in the season. When the highest number of reported influenza cases occurred in two or more weeks in a season, the peak was defined as the central week in the three-week period (or five-, seven-, etc. as needed) with the highest number of reported influenza cases. For example, if the number of cases in four consecutive weeks was 13-19-19-15, the peak was the third week, because this week had the highest number of cases and because 15 is higher than 13. The number of times the primary peak of the influenza epidemic occurred in each month of the year was determined for each country and for the Southern hemisphere, Northern hemisphere, and inter-tropical belt. The mean proportion of influenza cases in each month was then calculated for each country by averaging across all available seasons.

The duration of the influenza epidemic in each country and season was calculated by using the following two statistics: (a) the maximum proportion of influenza cases that occurred during a period of three consecutive months, and (b) the minimum number of months during which there were at least 80% of all influenza cases in the season. The median and interquartile range of these two statistics were then calculated for each country and for all countries in the Southern hemisphere, the Northern hemisphere, and the inter-tropical belt.

The timing of influenza A and B epidemics was compared using a restricted database including only those seasons with ≥ 50 reported cases of both influenza A and B and where both influenza types accounted for ≥ 20% of all influenza cases during the season. In such seasons, we calculated the number of times the peak of influenza A preceded, coincided, or followed that of influenza B, and the number of months between the peak of influenza A and that of influenza B. We then compared the median duration of influenza epidemics in seasons where both virus types circulated (i.e. where both virus types accounted for at least 20% of all influenza cases) and seasons where only influenza A circulated (i.e. where influenza B accounted for < 20% of all influenza cases during the season) by applying the Wilcoxon ranksum test.

## Results

### Influenza cases and seasons

The analysis included 212 influenza seasons during 2000–2014: 45 were in the seven countries in the Southern hemisphere, 87 were in the 17 countries in inter-tropical belt, and 80 were in the ten countries in the Northern hemisphere (Table 1). The number of influenza seasons per country ranged from two (Brazil West Central) to 12 (New Zealand, Portugal), with a median of five. The database included a total of 571,817 influenza cases. The median % of all cases in a season that were caused by influenza B was 23.4% in the Southern hemisphere (inter-quartile range [IQR] 9.5%-34.5%), 30.9% in the inter-tropical belt (IQR 12.2%-45.7%), and 24.5% in the Northern hemisphere (IQR 6.3%-40.6%),

**Table 1:** Influenza cases reported to the national influenza surveillance system by each participating country (from southern- to northern-most) and percentages of cases due to influenza type B virus.

Country	Latitude	Population (million)	No. seasons ^(1)^	No. influenza cases	% influenza B
***Southern hemisphere***			***45 (2000–2013)***	***143*,*102***	***23*.*3***
**New Zealand**	41.8 S	4.5	12 (2000–2012)	12,729	23.2
**Chile**	35.8 S	16.6	4 (2008–2012)	7,039	16.0
**Argentina (Santa Fe)**	31.4 S	3.2	3 (2010–2012)	664	18.7
**South Africa**	29.0 S	53.0	4 (2010–2013)	1,253	45.1
**Australia**	25.8 S	23.4	11 (2001–2012)	120,193	23.5
**Brazil South**	25.2 S	27.4	7 (2004–2012) ^(2)^	861	30.1
**Brazil Southeast**	23.3 S	80.4	4 (2008–2012)	363	33.3
***Inter-tropical belt***			***87 (2002–2014)***	***42*,*908***	***38*.*1***
**Madagascar**	19.4 S	21.3	11 (2002–2013)	2,638	40.2
**Brazil West Central**	15.5 S	13.3	2 (2006–2012) ^(2)^	152	12.5
**Brazil Northeast**	12.6 S	53.1	5 (2004–2012) ^(2)^	512	31.1
**Brazil North**	3.1 S	15.0	3 (2010–2012)	205	27.3
**Ecuador**	2.0 S	15.4	4 (2011–2014)	1,872	17.6
**Indonesia**	1.7 S	237.4	5 (2003–2007)	4,231	35.1
**Kenya**	0.4 S	44.4	5 (2007–2012)	4,700	25.0
**Singapore**	1.2 N	5.4	5 (2007–2012)	6,859	31.5
**Cameroon**	5.7 N	22.5	4 (2010–2013)	606	37.6
**Ivory Coast**	7.6 N	23.2	5 (2007–2012)	1,260	39.3
**Panama**	8.6 N	3.7	5 (2008–2013)	921	26.3
**Costa Rica**	10.0 N	4.6	3 (2010–2012)	2,185	19.9
**Nicaragua**	12.9 N	6.1	6 (2007–2013)	3,273	23.0
**El Salvador**	13.8 N	6.1	7 (2006–2013)	1,375	35.6
**Honduras**	14.8 N	8.2	3 (2010–2012)	797	19.6
**Guatemala**	15.7 N	15.4	7 (2006–2013)	2,093	18.9
**Viet Nam**	16.7 N	89.7	7 (2006–2013)	9,229	72.4
***Northern hemisphere***			***80 (2000–2014)***	***385*,*897***	***27*.*9***
**Bhutan**	27.4 N	0.7	3 (2010–2012)	690	33.6
**China South**	31.1 N	969.4	5 (2006–2011)	46,835	44.1
**Morocco**	32.0 N	33.2	7 (2003–2013) ^(2)^	1,130	21.4
**Turkey**	39.0 N	76.7	4 (2006–2010)	834	48.9
**Portugal**	39.3 N	10.4	12 (2000–2013)	3,684	25.5
**China North**	39.5 N	370.9	6 (2005–2011)	27,726	43.2
**Italy**	42.9 N	59.9	9 (2002–2011)	8,202	19.1
**USA**	45.6 N	317.6	11 (2000–2011)	289,413	23.9
**Kazakhstan**	48.0 N	17.9	4 (2010–2014)	1,195	23.8
**Ukraine**	49.1 N	44.6	10 (2000–2012) ^(2)^	1,335	23.3
**England**	52.3 N	53.0	9 (2003–2012)	4,853	32.0

^(1)^ The 2009 influenza season (2009–2010 in Northern hemisphere) was not included in the analysis.

^(2)^ We did not include the following twelve seasons because <50 influenza cases were reported: 2005 in Brazil South; 2007, 2008, 2010, and 2011 in Brazil West Central; 2005, 2007, and 2008 in Brazil Northeast; 2005–2006 and 2007–2008 in Morocco; and 2001–2002 and 2003–2004 in Ukraine.

### Timing of influenza epidemics in each region

**Fig 1:**
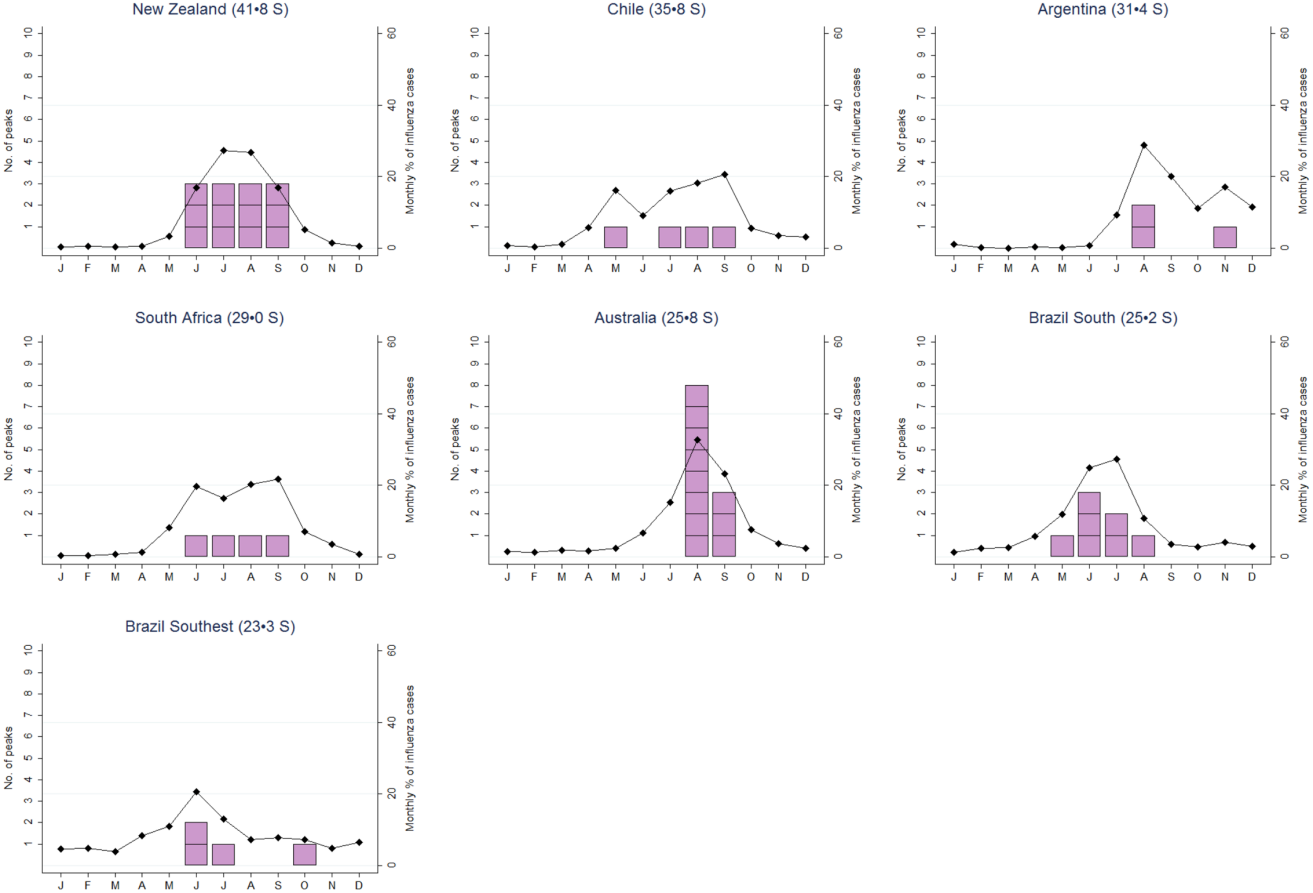
Mean percentage of influenza cases by month (black diamonds) and number of times the peak of the influenza season took place in each month (pink squares) for countries in the Southern hemisphere.

When averaged over time, influenza cases peaked between June and September in 41 of 45 influenza seasons in countries in the Southern hemisphere. The maximum monthly percentage of influenza cases was > 20% in all countries and > 30% in Australia (Fig 1).

**Fig 2:**
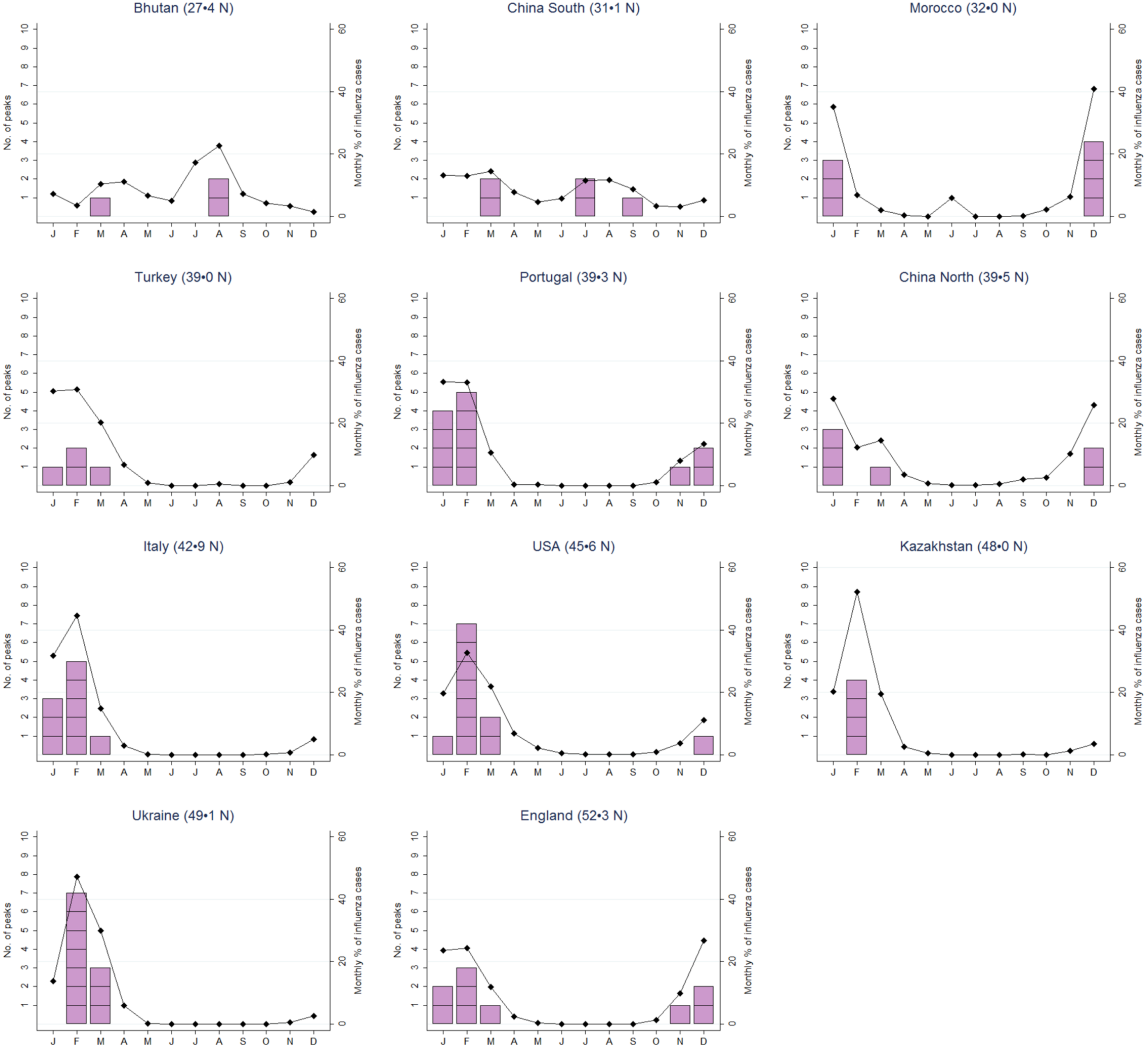
Mean percentage of influenza cases by month (black diamonds) and number of times the peak of the influenza season took place in each month (pink squares) for countries of Northern hemisphere.

In the eight northernmost countries of the Northern hemisphere, the peak of influenza activity was between December and March for 70 of 72 influenza seasons (Fig 2). The maximum monthly percentage of influenza cases was 25–30% for China North and England, 30–40% for Portugal, Turkey and the USA, and >40% for Italy, Kazakhstan, Morocco, and Ukraine. On the other hand, the seasonal peaks for the two southernmost countries of the Northern hemisphere were spread over six months (Bhutan) and over seven months (China South), and the maximum monthly percentage of influenza cases was < 15% in China South.

**Fig 3:**
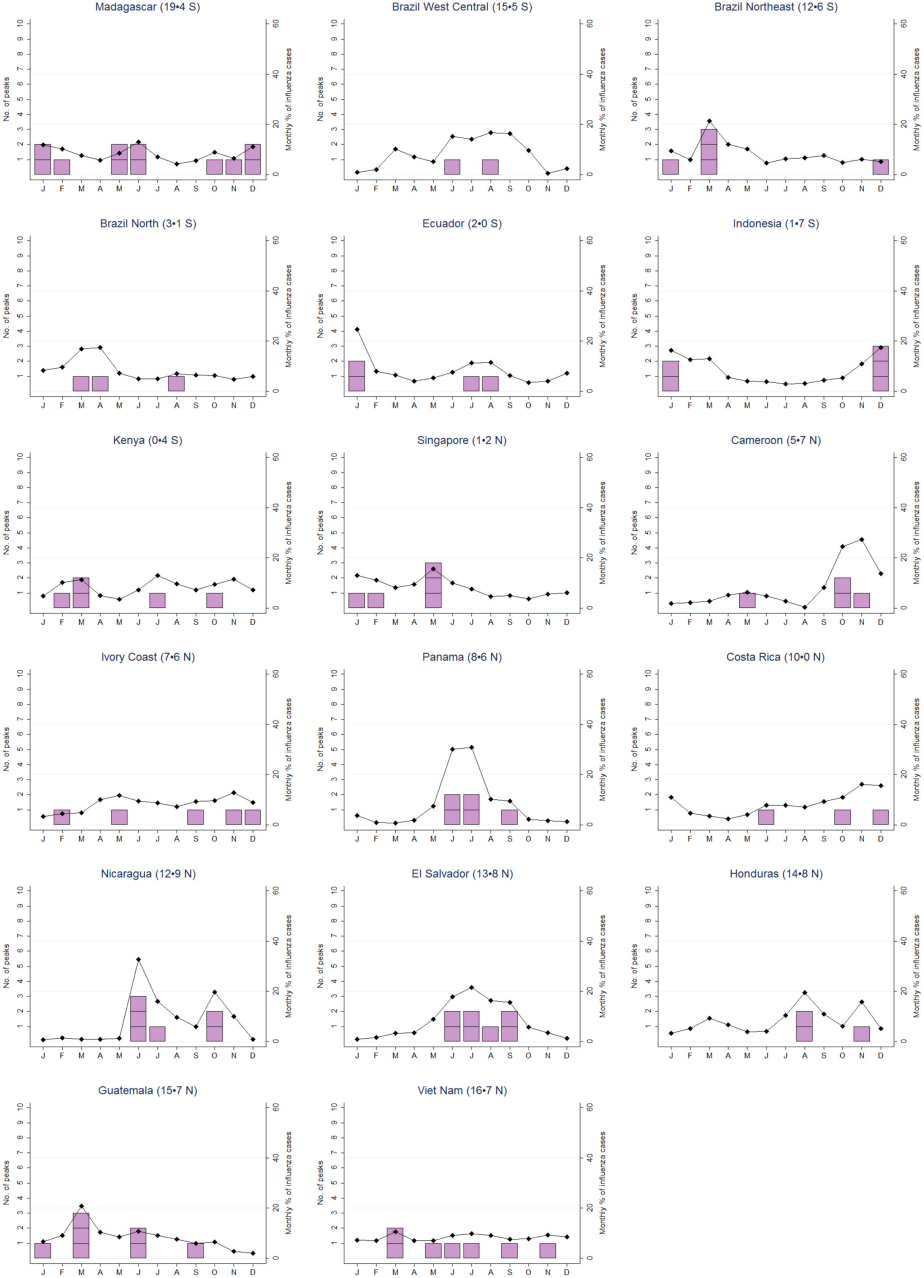
Mean percentage of influenza cases by month (black diamonds) and number of times the peak of the influenza season took place in each month (pink squares) for countries in the inter-tropical belt.

In the inter-tropical belt, the annual peaks tended to be spread over six months or longer in several countries (e.g., Madagascar, Kenya, Ivory Coast, Guatemala, and Viet Nam), although in some cases, the seasons were spread over less than six months (e.g., Indonesia, Panama, and El Salvador) (Fig 3). The maximum monthly percentage of influenza cases was <15% in four countries, 15–20% in seven countries, 20–25% in three countries, and >25% in three countries (Cameroon, Panama, and Nicaragua).

### Duration of seasonal influenza seasons and impact of co-circulation

Among temperate countries, a median of ≥70% of influenza cases occurred during the three-month peak, and at least 80% of influenza were included in a median of four or less consecutive months, for all countries except South Africa (62.1%, five months) and Brazil Southeast (41.7%, seven months) in the Southern hemisphere and Bhutan (65.5%, seven months) and China South (39.8%, nine months) in the Northern hemisphere (Table 2). Among countries of the inter-tropical belt, this was only the case for Panama and Nicaragua, while the median percentage of influenza cases during the three-month peak was as low as 34.6% in Viet Nam, and the number of consecutive months to have at least 80% of influenza cases in the season was ten in Kenya.

**Table 2:** Median percentage of influenza cases that occurred during the 3-month peak period and median number of months to have ≥80% of influenza cases during a season in countries of the Southern hemisphere, the inter-tropical belt, and the Northern hemisphere.

Country	Latitude	% of influenza cases during 3-month peak (median)	No. of consecutive months to have ≥80% of all influenza cases (median)
***Southern hemisphere***		***76*.*1***	***4***
**New Zealand**	41.8 S	85.0	3
**Chile**	35.8 S	79.4	4
**Argentina (Santa Fe)**	31.4 S	83.8	3
**South Africa**	29.0 S	62.1	5
**Australia**	25.8 S	74.5	4
**Brazil South**	25.2 S	71.7	4
**Brazil Southeast**	23.3 S	41.7	7
***Inter-tropical belt***		***54*.*9***	***7***
**Madagascar**	19.4 S	49.2	8
**Brazil West Central**	15.5 S	53.5	6
**Brazil Northeast**	12.6 S	37.9	9
**Brazil North**	3.1 S	46.1	9
**Ecuador**	2.0 S	48.8	7
**Indonesia**	1.7 S	56.2	9
**Kenya**	0.4 S	40.7	10
**Singapore**	1.2 N	40.6	9
**Cameroon**	5.7 N	73.5	5
**Ivory Coast**	7.6 N	54.5	8
**Panama**	8.6 N	84.0	3
**Costa Rica**	10.0 N	56.0	9
**Nicaragua**	12.9 N	82.6	3
**El Salvador**	13.8 N	64.5	5
**Honduras**	14.8 N	56.5	7
**Guatemala**	15.7 N	56.7	7
**Viet Nam**	16.7 N	34.6	9
***Northern hemisphere***		***91*.*0***	***3***
**Bhutan**	27.4 N	65.5	7
**China South**	31.1 N	39.8	9
**Morocco**	32.0 N	93.7	2
**Turkey**	39.0 N	80.8	3
**Portugal**	39.3 N	97.8	2
**China North**	39.5 N	78.1	4
**Italy**	42.9 N	94.5	2
**USA**	45.6 N	83.3	3
**Kazakhstan**	48.0 N	92.1	3
**Ukraine**	49.1 N	97.4	2
**England**	52.3 N	90.8	3

**Fig 4:**
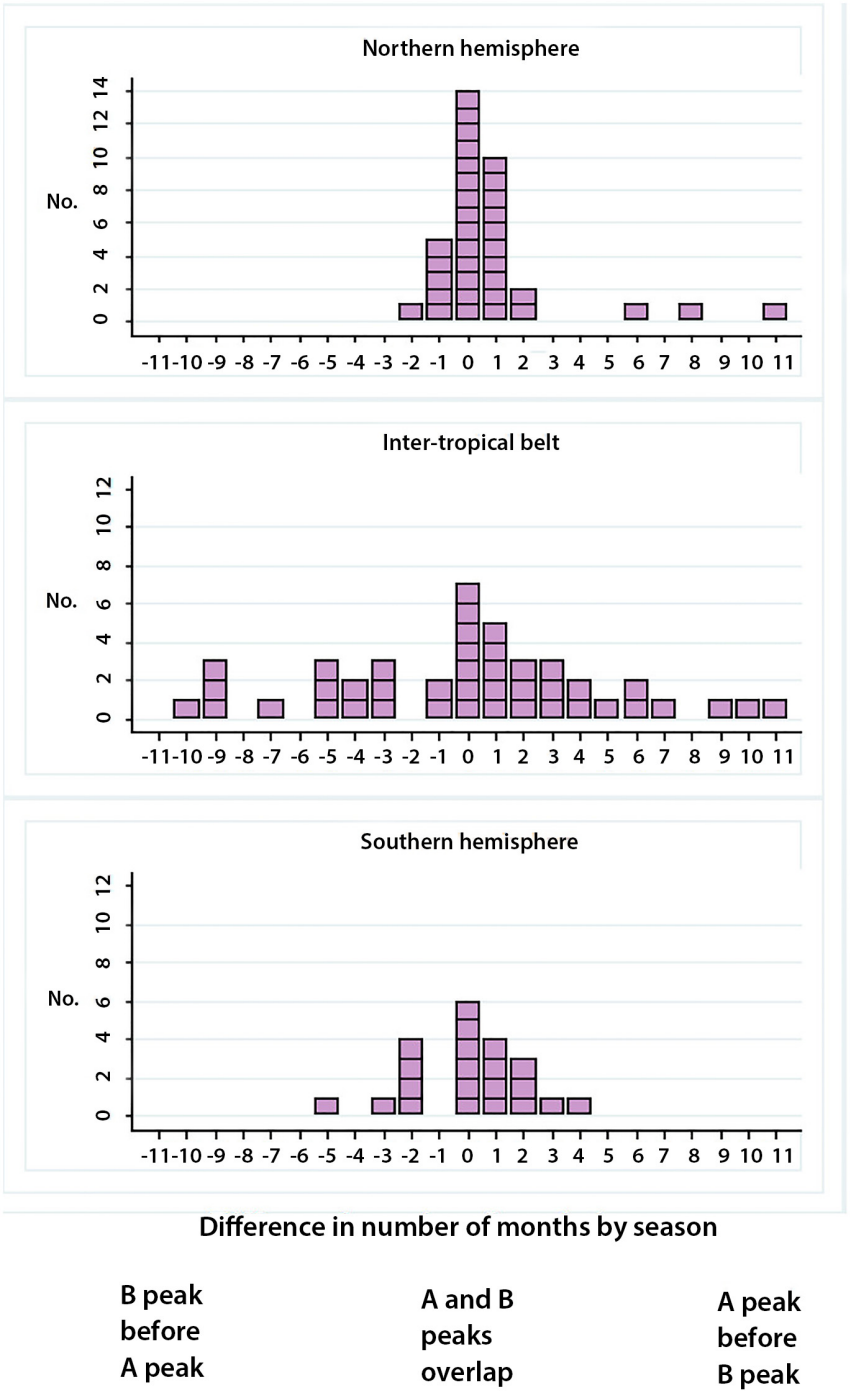
Distribution of influenza A and B peaks in the same season: Northern hemisphere, Inter-tropical belt and Southern hemisphere.

In the Southern hemisphere, the influenza A peak preceded the influenza B peak in nine seasons (43%), coincided with it in six seasons (29%), and followed it in six seasons (29%); in the inter-tropical belt, influenza A preceded influenza B in 20 seasons (48%), coincided with it in seven (16%) seasons, and followed it in fifteen seasons (36%); and in the Northern hemisphere, the influenza A peak preceded the influenza B peak in 15 seasons (43%), coincided with it in 14 seasons (40%), and followed it in six seasons (17%). The peaks of influenza A and B differed by three months or more in four of 21 seasons (19%) in the Southern hemisphere, in 25 of 42 seasons (60%) in the inter-tropical belt, and in three of 35 seasons (9%) in the Northern hemisphere (Fig 4).

The influenza season tended to be shorter when (only) influenza A circulated than when influenza A and B co-circulated (Table 3), a difference that was more pronounced in the inter-tropical belt (median proportion of influenza cases during the three-month peak, 64.5% for A seasons vs. 46.3% for A + B seasons) than in the Southern hemisphere (83.9% for A seasons vs. 73.6% for A + B seasons) and the Northern hemisphere (93.7% for A seasons vs. 85.9% for A + B seasons).

**Table 3:** Median percentage of influenza cases that occurred during the 3-month peak period and median number of months to have ≥80% of influenza cases during a season by zone (Southern hemisphere, inter-tropical belt, Northern hemisphere) and proportion of influenza B.

Zone	Season type	No. seasons	No. of months to have ≥ 80% of influenza cases during a season			% of influenza cases during 3-month peak		
			median	IQR	p-value ^(a)^	median	IQR	p-value ^(a)^
**Southern hemisphere**	**A**	18	3	3–4	0.010	83.9	76.6–89.9	0.004
	**A + B**	21	4	4–5		73.6	63.7–77.0	
**Inter-tropical belt**	**A**	31	7	4–8	0.054	64.5	48.1–79.6	0.002
	**A + B**	42	8	6–10		46.3	39.9–57.1	
**Northern hemisphere**	**A**	33	2	2–3	0.007	93.7	89.5–97.6	0.028
	**A + B**	35	3	3–4		85.9	80.0–94.5	

Seasons where influenza type A accounted for ≥ 80% of all influenza cases and with ≥ 50 reported cases of influenza A were considered influenza A seasons. Seasons where influenza type B accounted for ≥20% of all influenza cases and with ≥ 50 reported cases of influenza A and influenza B were considered influenza A + B seasons. IQR, interquartile range.

^(a)^ Wilcoxon ranksum test for comparison of medians.

## Discussion

This study showed that, between 2000 and 2014, the distribution of influenza cases was flatter, seasonal influenza activity lasted longer, and the peaks of influenza A and B coincided less frequently in tropical countries than in temperate countries. We also found that influenza seasons were longer when A and B viruses co-circulated and that this was especially marked in the tropics. Despite these general patterns, however, temporal characteristics of influenza epidemics in the tropics were highly heterogeneous, with a distinct seasonality observed in only a few countries.

Understanding the temporal characteristics of influenza epidemics is essential for planning influenza vaccination programs because vaccine effectiveness wanes over time [11, 19–20]. This waning effectiveness may be due to changes in circulating virus strains or because of a real decline in immunity, especially in the elderly. This means that the effectiveness of a vaccination campaign can be low when this is out of phase with the peak of the upcoming influenza season or in case of longer influenza seasons, which is a much more common occurrence in tropical compared to temperate countries. Accordingly, a detailed description of the timing of influenza epidemics over several consecutive seasons is crucial, especially in countries of the inter-tropical belt.

As expected, most influenza cases in the Southern and Northern hemispheres occur during a short period (2–4 months) in the winter months, although there are some exceptions like Bhutan, China South, and Brazil Southeast, which neighbour the inter-tropical belt. As the timing of epidemics does not substantially differ for influenza A and B, the duration of the influenza season is only marginally affected by circulation of influenza A and B viruses in temperate countries. In these countries, the optimal time to vaccinate can be determined with good accuracy, so if the vaccine and circulating strains are well matched, vaccination campaigns are expected to be effective. In countries of the inter-tropical belt, in contrast, the seasonal influenza peak can occur almost any time and generally accounts for fewer of the influenza cases. In fact, in some countries like the Ivory Coast and Viet Nam, the seasonal peak was almost imperceptible, and the term “peak” may even be inappropriate. Influenza seasons can last much longer in countries in this region than in other countries (possibly because of more strains that circulate together compared to temperate countries), and the different virus types tend to circulate more or less independently, complicating choices of the optimal time for vaccination.

Because of these limitations, it may be very difficult to establish an optimal time to vaccinate in tropical countries. In this region, the timing of influenza vaccination may be based on other considerations, such as seasonal crowding for social or religious reasons or organizational needs of local health systems. Alternatively, the recommendation may be to simply vaccinate at any time of the year [12]. In fact, in countries where influenza circulates year-round, vaccinating twice per year may be worth considering [7], at least for at-risk groups.

Only some of the countries in the inter-tropical belt are however characterized by a complete lack of seasonality of the influenza season. In particular, the temporal characteristics of influenza epidemics may differ between countries in the same geographical area and even between different regions within large countries [12, 21–22]. This is the case in Central America, a relatively small area with several countries stretched across fewer than ten degrees of latitude between the Tropic of Cancer and the Equator. For example, Panama (June to July) and El Salvador (June through September) show clear seasonality of influenza epidemics. Also, there appears to be a seasonal peak in Guatemala (March) and the influenza season seems to extend between June and January in Costa Rica. Two countries, Nicaragua and Honduras, appear to have secondary influenza peaks.

The temporal characteristics of influenza epidemics in countries of the inter-tropical belt are the result of a complex and still poorly understood interaction of several climatic and ecological drivers, including temperature, altitude, humidity, precipitation, and population density [23–24]. To develop recommendations on the optimal time to vaccinate, time-series analyses of influenza epidemics from previous seasons should be conducted with a fine enough resolution to allow adaptation for these factors, some of which can vary substantially, even within a few hundred kilometres. This is especially important for countries in the inter-tropical belt, because of the highly heterogeneous temporal patterns of influenza epidemics in this world area [25–29], although it may also be relevant for large countries in other regions [9].

Our analysis included more than 570,000 influenza cases over 14 years in 30 countries for a total of 212 influenza seasons. Although this is a rich source of data, comparing the data between countries may be complicated by the substantial differences between the national influenza surveillance systems [15]. In particular, the proportion of influenza cases reported by each surveillance system may differ greatly. For example, reporting differs between Australia and Madagascar, even though they have comparable populations and the same number of influenza seasons included in this analysis. Differences in reporting may also have been present within a single country over time, for example, before and after the 2009 influenza H1N1 pandemic. We used “season” as the unit of analysis to accommodate for this limitation, as this gives each “season” an equal weighting and the results are not weighted towards the post-pandemic period when most of the data are available. It was not possible to define the influenza activity peak in terms of the percentage of respiratory specimens testing positive for influenza, as this information was not available for some of the countries participating to the GIBS. Another potential limitation is that only a few seasons were included for some countries (for example, only three seasons were included for Argentina, Brazil North, Costa Rica, Honduras and Bhutan, and only two for Brazil West Central), so that chance may explain some of the country-specific differences we observed. We would have greatly benefited from having data from a much longer period for all participating countries, both because this may be needed to determine the full period of influenza activity and define whether some seasonality exists, especially in countries in the inter-tropical belt, and also in order to avoid the over-representation of countries that contribute data for a large number of seasons. Finally, although regional data were available for Brazil and China, the analysis could have benefitted from more regional data, especially for large countries.

In conclusion, we found that the temporal characteristics of influenza epidemics differ greatly between the Southern and Northern hemispheres and the inter-tropical belt, and more importantly, even between countries within the inter-tropical belt. Based on these results, a harmonized timing of vaccination, similar to the Northern and Southern hemisphere, would probably not be very effective for tropical countries, and we recommend that a local approach be adopted instead, with each country developing its own national influenza vaccination policy based on the analysis of epidemiological data collected locally. This will only be possible however if community- and hospital- based sentinel surveillance systems are set up and run for several years. Reliable surveillance data are not yet available for many countries in the inter-tropical belt, although there are some excellent exceptions [12, 30–32]. Filling this gap should be a priority to allow vaccine recommendations in the tropics to be based on the analysis of epidemiological and virological data collected at a local level rather than educated guesses.

## Supporting Information

S1 File Contact information of data owners. (DOC)
