# Correction: Temporal Patterns of Influenza A and B in Tropical and Temperate Countries: What Are the Lessons for Influenza Vaccination?

**DOI:** 10.1371/journal.pone.0155089

**Published:** 2016-05-02

**Authors:** S Caini, W Andrade, S Badur, A Balmaseda, A Barakat, A Bella, A Bimohuen, L Brammer, J Bresee, A Bruno, L Castillo, MA Ciblak, AW Clara, C Cohen, C Daouda, C de Lozano, D De Mora, K Dorji, GO Emukule, RA Fasce, L Feng, WA Ferreira de Almeida, R Guiomar, JM Heraud, O Holubka, QS Huang, HA Kadjo, L Kiyanbekova, H Kosasih, G Kusznierz, VJM Lee, J Lara, M Li, L Lopez, Hoang PV Mai, Henriques CM Pessanha, ML Matute, A Mironenko, B Moreno, JA Mott, R Njouom, Nurhayati, A Ospanova, R Owen, R Pebody, K Pennington, S Puzelli, MT Quynh Le, NH Razanajatovo, A Rodrigues, JM Rudi, M Venter, MA Vernet, A L Wei, S Wangchuk, J Yang, H Yu, M Zambon, F Schellevis, J Paget, Global Influenza B Study

Dr. Jeffery Cutter and Dr. Raymond Tzer Pin Lin should be removed from the author byline and included in the Group Authorship. Dr. Vernon Jian Ming Lee should be included in the author byline and listed as the thirty-first author. His affiliation is 13: Communicable Diseases Division, Ministry of Health, Singapore, Singapore. Dr. Ang Li Wei should be included in the author byline and listed as the fifty-fourth author. His affiliation is with Epidemiology and Diseases Control Division, Ministry of Health, Singapore, Singapore.
