# Supplementary material for: Temporal Patterns of Influenza A and B in Tropical and Temperate Countries: What Are the Lessons for Influenza Vaccination?
Source: PLoS One. 2016 Mar 31;11(3):e0152310. doi: 10.1371/journal.pone.0152310 (PMC4816507; doi:10.1371/journal.pone.0152310)
Supplement: S1 File — (DOC) [file pone.0152310.s001.doc]

These datasets are owned by the participating countries, and thus cannot be shared publicly by the study group at NIVEL. Researchers interested in obtaining the country-specific datasets may contact the individuals listed in the Supporting Information for further details regarding data access.

**Argentina (Santa Fe Province)**: Gabriela Kusznierz ([labconi@yahoo.com.ar](mailto:labconi@yahoo.com.ar))

**Australia**: Rhonda Owen ([rhonda.owen@health.gov.au](mailto:rhonda.owen@health.gov.au))

**Bhutan**: Sonam Wangchuk ([swangchuk@health.gov.bt](mailto:swangchuk@health.gov.bt))

**Brazil**: Jose Ricardo Pio Marins ([ricardo.marins@saude.gov.br](mailto:ricardo.marins@saude.gov.br))

**Cameroon**: Guy Vernet ([vernet@pasteur-yaounde.org](mailto:vernet@pasteur-yaounde.org))

**Chile**: Rodrigo Fasce ([rfasce@ispch.cl](mailto:rfasce@ispch.cl))

**China**: Feng Luzhao ([fenglz@chinacdc.cn](mailto:fenglz@chinacdc.cn))

**Costa** **Rica**: Alexey W. Clara ([wclara@cdc.gov](mailto:wclara@cdc.gov))

**Ecuador**: Alfredo Bruno ([alfredobruno@yahoo.es](mailto:alfredobruno@yahoo.es))

**El** **Salvador**: Alexey W. Clara ([wclara@cdc.gov](mailto:wclara@cdc.gov))

**England**: Maria Zambon ([maria.zambon@phe.gov.uk](mailto:maria.zambon@phe.gov.uk))

**Guatemala**: Alexey W. Clara ([wclara@cdc.gov](mailto:wclara@cdc.gov))

**Honduras**: Alexey W. Clara ([wclara@cdc.gov](mailto:wclara@cdc.gov))

**Indonesia**: Herman Kosasih ([herman_kosasih@yahoo.com](mailto:herman_kosasih@yahoo.com))

**Italy**: Caterina Rizzo ([caterina.rizzo@iss.it](mailto:caterina.rizzo@iss.it))

**Ivory** **Coast**: Herve A. Kadjo ([rvkdjo@yahoo.fr](mailto:rvkdjo@yahoo.fr))

**Kazakhstan**: Gulzhan Muratbayeva ([hnv2@cdc.gov](mailto:hnv2@cdc.gov))

**Kenya**: Joshua Mott ([zud9@cdc.gov](mailto:zud9@cdc.gov))

**Madagascar**: Jean-Michel Heraud ([jmheraud@pasteur.mg](mailto:jmheraud@pasteur.mg))

**Morocco**: Amal Barakat ([amal.barakat@yahoo.fr](mailto:amal.barakat@yahoo.fr))

**New** **Zealand**: Sue Huang ([sue.huang@esr.cri.nz](mailto:sue.huang@esr.cri.nz))

**Nicaragua**: Alexey W. Clara ([wclara@cdc.gov](mailto:wclara@cdc.gov))

**Panama**: Alexey W. Clara ([wclara@cdc.gov](mailto:wclara@cdc.gov))

**Portugal**: Balthazar Nunes ([baltazar.nunes@insa.min-saude.pt](mailto:baltazar.nunes@insa.min-saude.pt))

**Singapore**: Vernon Lee ([vernonljm@hotmail.com](mailto:vernonljm@hotmail.com))

**South** **Africa**: Marietjie Venter ([yds8@cdc.gov](mailto:yds8@cdc.gov))

**Turkey**: Meral Akcay Ciblak ([ciblakm@yahoo.com](mailto:ciblakm@yahoo.com))

**Ukraine**: Alla Mironenko ([miralla@ukr.net](mailto:miralla@ukr.net))

**USA**: Joseph Bresee ([jsb6@cdc.gov](mailto:jsb6@cdc.gov))

**Viet** **Nam**: Le Thi Quinh Mai ([lom9@hotmail.com](mailto:lom9@hotmail.com))
